# Synthesis, characterization and thermal decomposition kinetics of a bio-based transparent nylon 10I/10T

**DOI:** 10.1080/15685551.2018.1543633

**Published:** 2018-11-14

**Authors:** Bingxiao Liu, Guosheng Hu, Jingting Zhang, Chunhui Fang

**Affiliations:** Institute of Macromolecules and Bioengineering, School of Materials Science and Engineering, North University of China, Taiyuan, China

**Keywords:** Bio-based, nylon, melt polymerization, thermal decomposition kinetics, activation energy (E)

## Abstract

Herein, we report a novel transparent engineering plastic nylon 10I/10T based on bio-based poly(decamethylene isophthalamide) (nylon10I). We have demonstrated the one-step melt polycondensation synthesis of transparent nylon and carried out Fourier transform infrared spectroscopy (FT-IR) and proton nuclear magnetic resonance (^1^H NMR) to confirm the chemical structure. Furthermore, the dynamic mechanical analysis (DMA) and thermogravimetric analysis (TGA) were used to analyze the thermal properties. Glass transition temperature (*T_g_*) and thermal decomposition onset temperature (*T_1_*) of nylon 10I/10T (15 wt. % 10T) were 118.9 and 438.0 °C, respectively. The intrinsic viscosity, water absorption, light transmittance, mechanical properties, solvent resistance and the decomposition mechanism of nylon 10I/10T have also been investigated. The results show that the nylon 10I/10T has lower water absorption and enhanced solvent resistance compared to nylon 6–3-T, which indicates that nylon 10I/10T is a promising transparent plastic.

## Introduction

The transparent nylon, a prominent member of polymer-family, offers an array of fascinating properties. Recently, it has garnered special research interest due to its excellent light transmittance, which is higher than polycarbonate (PC) and similar to poly(methyl methacrylate) (PMMA), superior solvent and abrasion resistance [1]. Superior solvent resistance is considered as a critical property for transparent materials, which are applied in aggressive media-conducting systems. Moreover, recent studies have demonstrated that transparent nylon can be used in a wide range of applications such as optical instruments, food packaging films and athletic equipment [2–4].

Based on the compositional difference, transparent nylon can be classified as fully aromatic transparent nylon, semi-aromatic transparent nylon and aliphatic-aromatic transparent nylon. The semi-aromatic transparent nylon (nylon10I/10T) has the advantages over aromatic transparent nylons, such as favorable melt processability, and aliphatic transparent nylons, such as superior thermal and mechanical properties [5,6]. It is worth noting that the commercially available semi-aromatic transparent nylon 6–3-T offers excellent transmittance. However, it renders obvious disadvantages, such as high water absorption and low alcohol resistance. In addition, the monomer materials for nylon 6–3-T, terephthalic acid (PTA) and 2,2,4/2,4,4-trimethylhexamethylene diamine, are both petrochemicals [7]. However, the rapidly depleting fossil resources, along with adverse environmental impacts, require that renewable bio-based materials should replace the petrochemicals from industrial synthesis processes [8–11].

Consequently, we aimed to replace the conventional petrochemicals with isophthalic acid (IPA) and castor-oil based decamethylenediamine (DA10) to obtain long chain nylon10I, which is a low-water absorption bio-based transparent nylon [12,13]. The PTA was added to improve the thermal stability and mechanical properties of nylon10I.

Wang et al. have synthesized semi-aromatic nylon block copolymer by low temperature solution polycondensation, which is not desirable due to the lower molecular weight of polymerization products, use of the corrosive solution and recovery of the reaction solution [14]. On the other hand, the solid state polycondensation used by Hasuo et al. [15] contains salt formation and polymerization, which makes the preparation process complicated and lengthy. Melt polymerization is one of the most commonly employed polymerization methods, which has high requirements for equipment and raw material ratio. Also, it does not need to recover the solution and exhibits shorter cycles compared to solution polycondensation and solid state polycondensation [16,17].

It is obvious that the synthesis of semi-aromatic transparent nylon from bio-based precursors has not been reported yet. Therefore, for the first time, we aimed to synthesize the transparent nylon 10I and copolymerized nylon10I/10T. The synthesized transparent nylon 10I/10T has been characterized by FT-IR and ^1^H NMR. The thermal behavior was investigated by DMA and TGA. The thermal degradation kinetics studies are essential for engineering scale-up and application of thermochemical conversion of polymer wastes to valuable chemicals.

Moreover, these studies are recommended by the International Confederation for Thermal Analysis and Calorimetry (ICTAC) [18,19], which are included in the present study. The kinetics parameters and activation energy were calculated by Kissinger-Akahira-Sunose (KAS), Flynn−Wall−Ozawa (FWO) and Tang methods. The degradation reaction mechanism was determined by the Coats–Redfern method. The intrinsic viscosity, water absorption, light transmittance, mechanical properties and solvent resistance of nylon10I/10T were also assessed and reported here.

## Experimental

### Materials and synthesis

The PTA (99.0%) was supplied by Beijing Yanshan Lithification Chemical Co. Ltd. (Beijing, China). The benzoic acid (BA) (99.5%) was purchased from Tianjin Shen Tai Chemical Reagent Co. Ltd. (Tianjin, China). The Wuxi Yinda Nylons Co. Ltd. (Wuxi, China) provided IPA (99.9%) and DA10 (99.2%).

**Figure 1.**
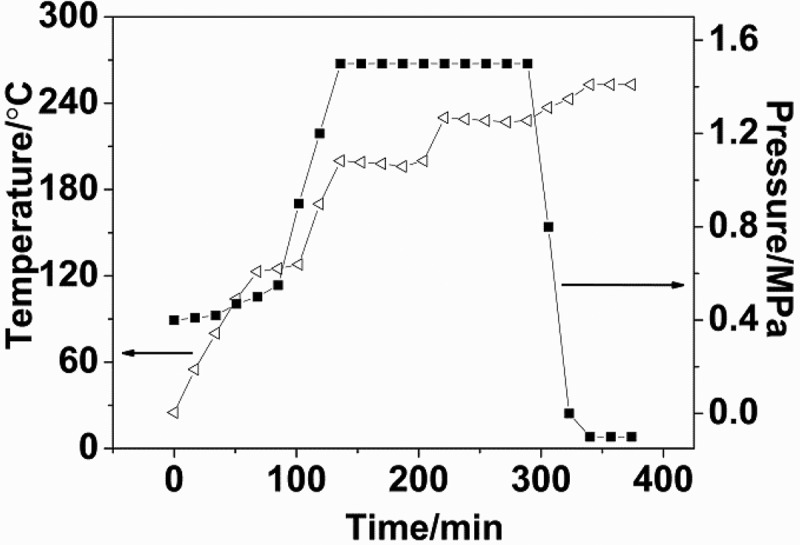
Changes in temperature and pressure during polymerization.

The melt condensation polymerization was performed in a three-step process, involving pre-polymerization, polycondensation and viscosity enhancement reaction. Figure 1 presents the change in temperature and pressure during polymerization. In a typical reaction, monomers, BA and distilled water were added in an autoclave. The detailed dosage is as presented in Table 1. Then, the autoclave was purged with N_2_ for 5 minutes. At a pressure of 0.4 MPa and temperature of 230 ℃, the mixture temperature remained at 125 ℃ until complete evaporation of water. This stage promotes the homogenization of the reaction mixture without any polymerization. After boiling, the temperature and pressure were rapidly increased. When the reaction temperature and pressure reached at 200 ℃ and 1.5 MPa, respectively, with 1 h of holding time, the heater set point was changed to 260 ℃ and the temperature was held constant at 230 ℃. After 1.5 h, the pressure of the autoclave was gradually released to atmospheric pressure and the reaction temperature of the sample was increased to 243 ℃. At the same time, the autoclave was evacuated to the pressure of −0.1 MPa and reaction was continued for another 0.5 h to obtain nylon 10I and nylon 10I/10T.

**Table 1.** Composition of nylon 10I and nylon 10I/10T copolymers.

Number	Sample	DA10(mol)	IPA(mol)	PTA(mol)	BA(mol)	H_2_O(mL)
A	nylon 10I	1.02	1.00	-	0.03	100
B	nylon 10I/10T(2.5%)	1.02	0.975	0.025	0.03	100
C	nylon 10I/10T(5%)	1.02	0.95	0.05	0.03	100
D	nylon 10I/10T(10%)	1.02	0.90	0.10	0.03	100
E	nylon 10I/10T(15%)	1.02	0.85	0.15	0.03	100

## Characterization

The intrinsic viscosity of the nylon 10I and nylon 10I/10T was measured by using Ubbelohde viscometer (model 1835). The nylons were dissolved in concentrated sulfuric acid. The FT-IR spectra were recorded on a Nicolet-Nexus-670 spectrometer. The ^1^H NMR spectra were recorded with a Bruker DPX-400 at 400 MHz, using deuterated trifluoroacetic acid as a solvent. The light transmittance test was carried out by using SDR850.

The samples of nylon 10I and nylon 10I/10T were prepared by injection molding for the mechanical properties evaluation. The tensile property, bending property and izod impact property were tested by Universal testing machine CMT6104 and Impact testing machine XC-2.75D. These tests were conducted according to the GB/T 1040–2006 (Chinese standard), GB/T 9341–2008 (Chinese standard) and ISO 178–2003, respectively.

The DMA was conducted by using METTLER SDTA861 in the bending mode, at 2.0 Hz, and samples were heated at 3 °C/min from 40 to 150 °C. The TGA was carried out by a Mettler Toledo equipment. The samples were heated in the temperature range of 25–700 ℃ at different heating rates, such as 5, 10, 20 and 30 ℃/min, under N_2_ environments. The gas flow rate was 50 mL/min.

## Results and discussion

### Synthesis of nylon 10I and nylon 10I/10T

We have synthesized nylon 10I and nylon 10I/10T from DA10, IPA and PTA by melt polycondensation (the reaction scheme is shown in Figure S1.). BA was added to control the molecular weight of nylons. At the same time, 100 mL of distilled water was added to obtain high vapor pressure and counter the volatile nature of diamine during the polymerization [20]. It should be noted that the autoclaves were purged with N_2_ before heating to remove the oxygen and ensure excellent temperature control.

The intrinsic viscosity values ([η]) of nylon 10I and nylon 10I/10T were measured by dissolving in concentrated sulfuric acid and the obtained results are listed in Table 2. We have observed that the intrinsic viscosity of nylon 10I and nylon 10I/10T ranged from 88.9 to 91.3 mL/g, which corresponds to the high molecular weight. In addition, the water absorption of nylon 10I and nylon 10I/10T ranged from 0.2 and 0.23% (Table 2), which is much lower than the water absorption of nylon 6–3-T (7.5%), which can be ascribed to the lower hydrophilic amino density in nylon 10I and nylon 10I/10T than nylon 6–3-T.

**Table 2.** The intrinsic viscosity and water absorption of samples.

Samples	A	B	C	D	E	nylon 6–3-T
[η] (mL/g)	90.9	89.6	91.0	91.3	88.9	-
Water absorption/%	0.20	0.21	0.22	0.22	0.23	7.5

### FT-IR spectra

**Figure 2.**
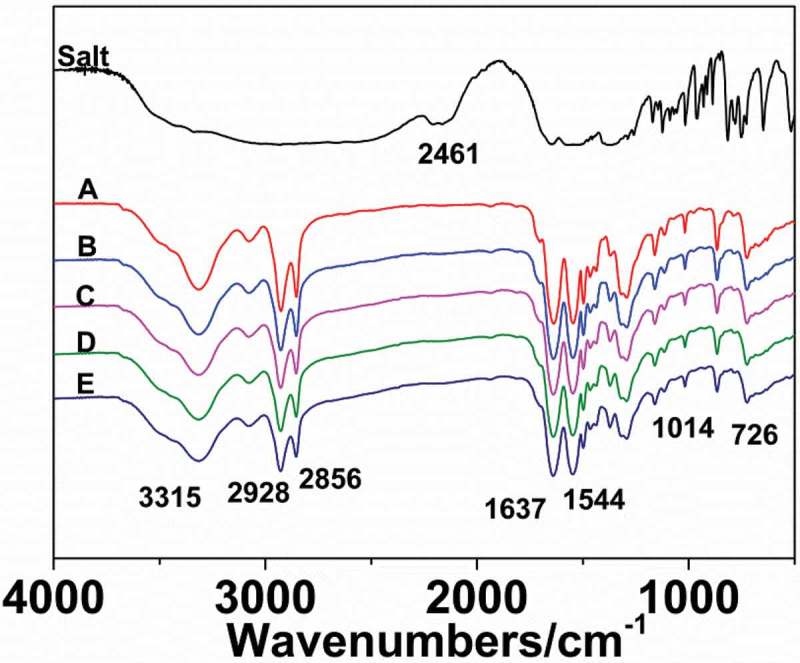
FT-IR spectra of nylon 10I/10T salt and nylon 10I/10T.

Figure 2 presents the FT-IR spectra of the nylon 10I/10T salt, nylon 10I and nylon10I/10T samples. As shown in Figure 2, the special peak of nylon 10I/10T salt appeared at 2461 cm^−1^ (NH_3_^+^, frequency doubling and combination band). The most prominent peaks of nylon 10I/10T, such as 3315 cm^−1^ (N-H stretching vibration), 2928 cm^−1^ and 2856 cm^−1^ (CH_2_, asymmetric and symmetric stretching vibration, respectively), 1637 cm^−1^ (amide I, C = O stretching vibration), 1544 cm^−1^ (amide II, C–N stretching and CO–N–H bending vibration), 1014 cm^−1^ (amide IV, C–CO stretching vibration) and 726 cm^−1^ (amide V, N-H out-of-plane bending vibration), can be indexed. All these characteristic absorption peaks are consistent with previous reports of nylons [21]. Also, it can be seen that the characteristic absorption peak at 2461 cm^−1^ (NH_3_^+^) has disappeared in the FT-IR spectra of nylon 10I/10T, which is consistent with the formation mechanism of nylon 10I/10T.

### ^1^H NMR analysis

**Figure 3.**
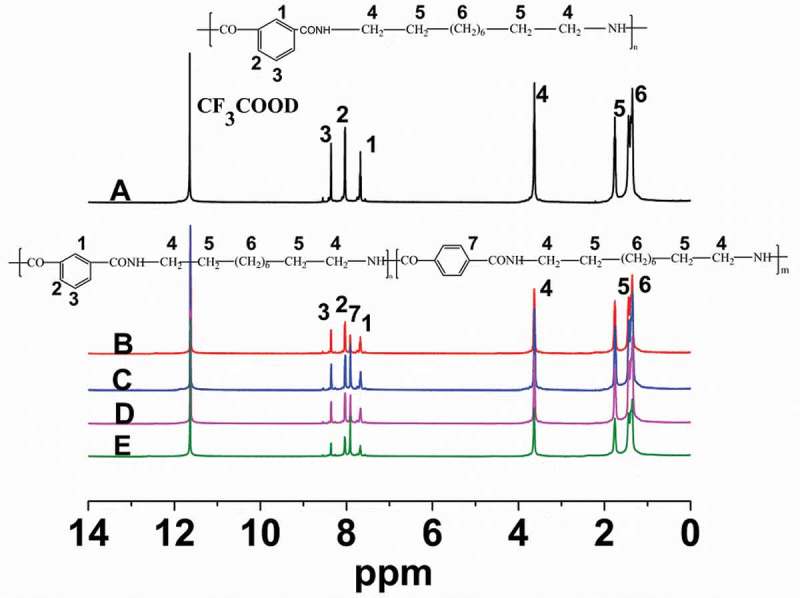
^1^ H NMR spectra of nylon 10I and 10I/10T.

In order to further characterize the structure of nylon 10I and nylon10I/10T, we have carried out ^1^H NMR analysis. The ^1^H NMR spectra of nylon 10I and nylon 10I/10T are depicted in Figure 3. The chemical shifts, in the range of 8.45–7.44 ppm, originate from aromatic protons (position 1, 2, 3). The chemical shifts at 3.71–3.69 ppm can be attributed to the protons of methylene adjacent to the NH group (position 4). The chemical shifts at 1.86–1.81 ppm and 1.50–1.43 ppm correspond to the protons at the position 5 and 6, respectively. The peak at 11.6 ppm originates from deuterated trifluoroacetic acid. The chemical shifts of nylon 10I/10T are similar to nylon 10I, except that the shifts in the range of 8.45–7.44 ppm correspond to position 1, 2, 3 and 7. One should note that position 7 corresponds to the proton on the benzene ring of PTA, which confirms the formation of nylon 10I/10T. The results of the ^1^H NMR and FT-IR spectra are in good agreement with the theoretical values of these compounds.

### Light transmittance test

**Figure 4.**
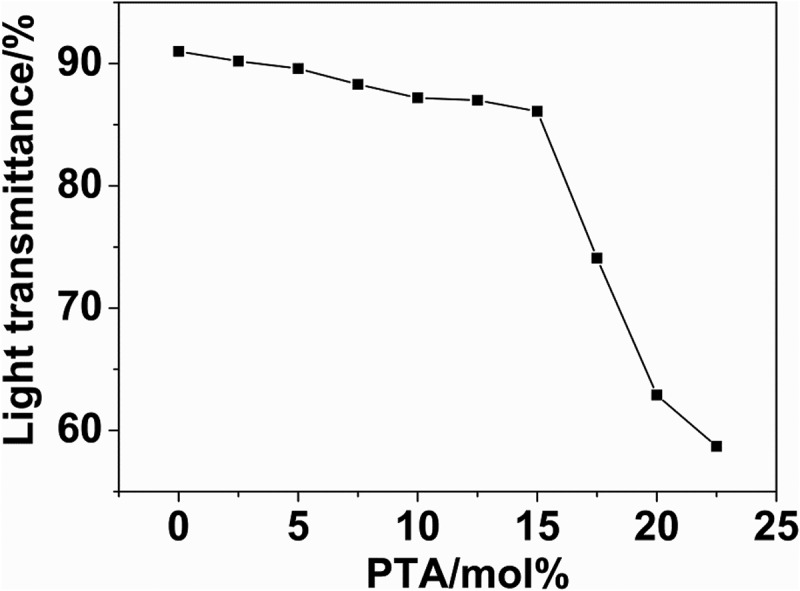
The light transmittance of nylon 10I/10T in the presence of various concentrations of PTA.

After structural validation, we have conducted a light transmittance test, which is one of the most important properties of transparent nylon. The light transmittance test results of nylon 10I and nylon 10I/10T are presented in Figure 4. One should note that after a threshold value of PTA content exceeds 15 mol%, the light transmittance of nylon 10I/10T dramatically decreased. Moreover, it has been observed that the effect of PTA additive on light transmittance is negligible compared to nylon 10I in the range of 0–15 mol%, which indicates that PTA did not influence the light transmittance in this range. This may be because pure nylon 10T is a crystalline polymer with a regular structure, but pure nylon-6–3T is an amorphous polymer. Therefore, the transparency of nylon 10I/10T is influenced when the content of nylon 10T exceeds a threshold limit in the nylon 10I/10T chain. In addition, the light transmittance of as-prepared nylons ranged from 85–90%, which is comparable to nylon 6–3-T (90%).

### Mechanical properties

To further elucidate the feasibility of the nylon10I/10T as a transparent plastic, we have assessed the mechanical properties. The mechanical performance parameters of the nylon 10I and nylon10I/10T are summarized in Table 3. It can be observed that there is an increase in the tensile strength and breaking elongation of the nylon10I/10T with the increase of PTA content. Moreover, other mechanical performance parameters of nylon 10I/10T, such as bending strength, bending modulus and izod impact strength, are comparable to nylon 10I. One should note that due to the addition of PTA, hydrogen bonds are more easily formed between molecules, resulting in enhanced intermolecular forces.

**Table 3.** The mechanical properties of nylon 10I and nylon 10I/10T.

Samples		Tensile strength[MPa]	Breaking elongation[%]	Bending strength[MPa]	Bending modulus[MPa]	Izod impact strength[KJ⸱m^2^]
A	60.3		69.1	47.4	2058	15.7
B	67.7		84.0	44.2	2063	14.0
C	69.3		93.0	44.6	2049	14.4
D	72.7		104.2	45.0	2053	13.9
E	76.3		108.0	45.9	2044	13.0

### Solvent resistance

The solvent resistance parameters of the nylon 10I and nylon 10I/10T were examined in the various organic solvents, including methanol, n-butyl alcohol, n-propyl alcohol, aniline, glacial acetic acid and N，N-dimethylformamide, and the results are summarized in Table 4. Clearly, the date confirm that the nylon 10I and nylon 10I/10T have better solvent resistance compared to nylon 6–3-T. This can be due to the lower hydrophilic amino density of nylon 10I and nylon 10I/10T than nylon 6–3-T. Hence, according to the similar dissolve mutually theory, nylon 6–3-T is easily dissolved in polar solvents due to the higher content of polar amide groups.

**Table 4.** The chemical resistance of nylon 10I, nylon 10I/10T and nylon 6–3-T.

solvent	nylon 10I	nylon 10I/10T	nylon 6–3-T
methanol	-	-	+
n-butyl alcohol	+ −	+ −	+
n-propyl alcohol	+ −	+ −	+
aniline	-	-	+
glacial acetic acid	+ −	+ −	+
N，N-dimethylformamide	-	-	+

+, soluble; + −, swelling; – insoluble

### DMA analysis

**Figure 5.**
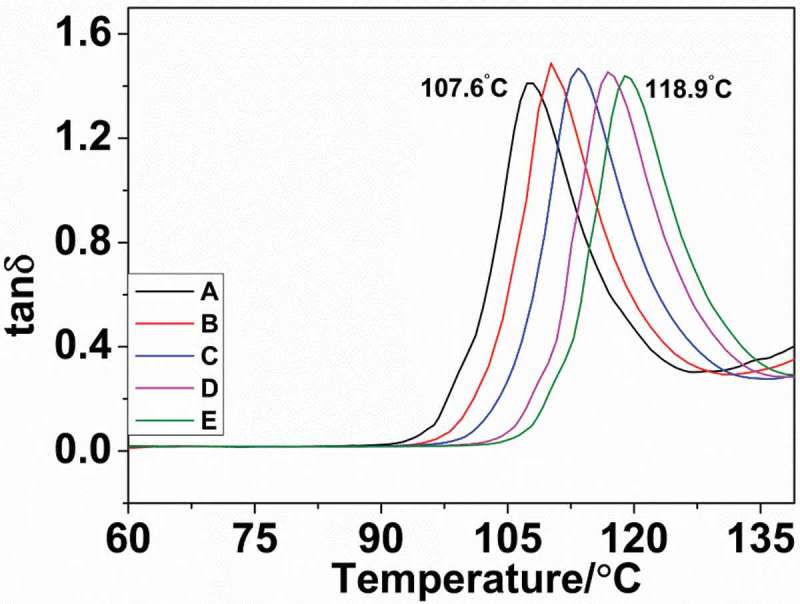
DMA curves of nylon 10I and nylon 10I/10T.

The results obtained from DMA analysis of nylon 10I and nylon10I/10T are presented in Figure 5. Intriguingly, the corresponding peak of tanδ represents the glass transition temperature (T_g_) [22,23]. Based on the given results, a notable increase in the *T_g_* of nylon 10I/10T with the increase of PTA has been observed, which can be assigned to the improved ability of intermolecular chains to form hydrogen bonds and restricted chain movement.

### Thermal degradation analysis

Various heating schemes, such as a single heating rate and multiple heating rates, can be utilized to investigate the kinetics of the thermal decomposition process. From the published literature, the methods using multiple heating rates are adopted for reliable results [24–26].

**Figure 6.**
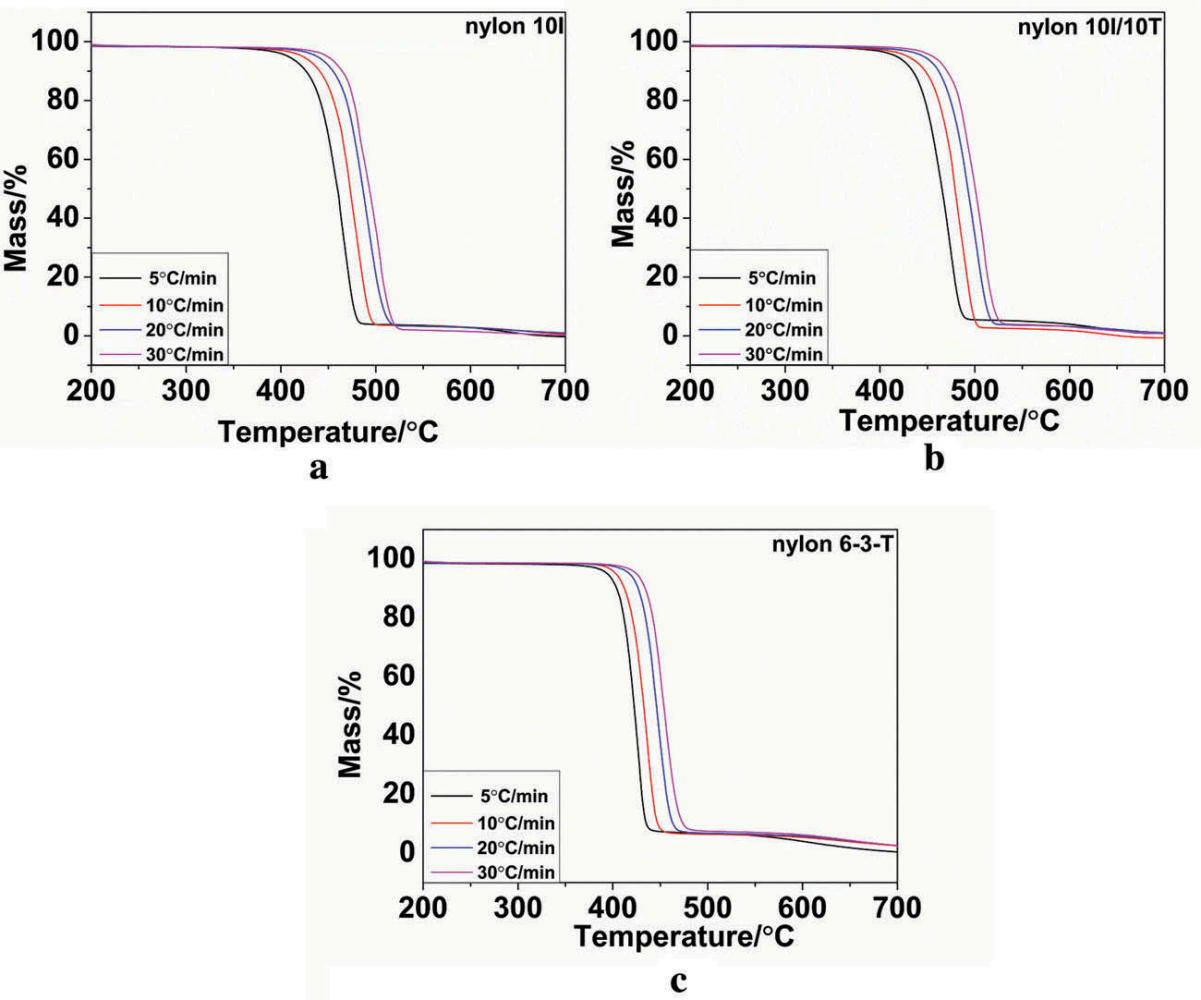
TGA curves: (a) nylon 10I, (b) nylon 10I/10T and (c) nylon 6–3-T.

**Figure 7.**
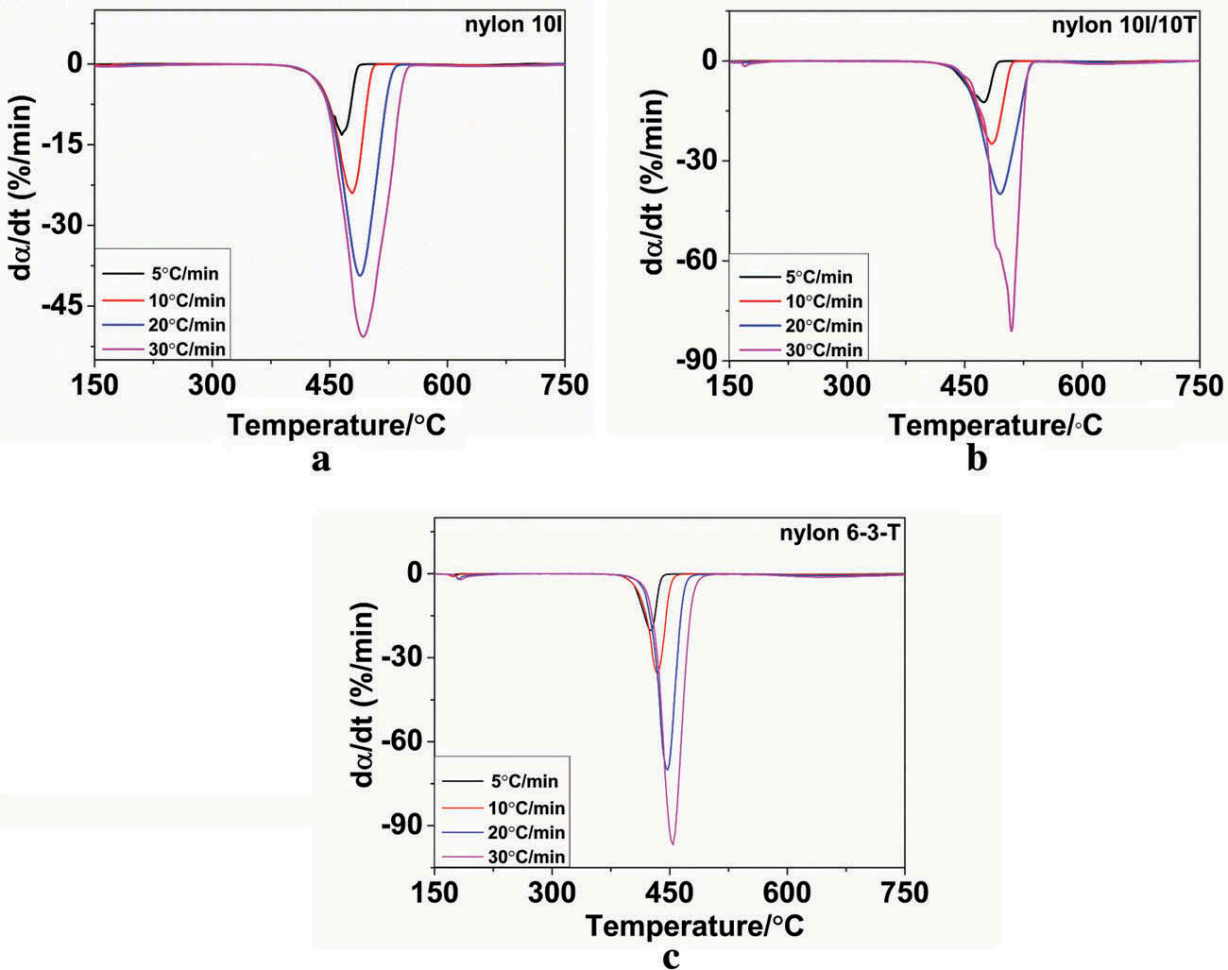
DTG curves: (a) nylon 10I, (b) nylon 10I/10T and (c) nylon 6–3-T.

The TGA and DTG curves under N_2_ were obtained, in the temperature range 40–800 ᵒC, at different heating rates of 5, 10, 20 and 30 ᵒC/min from nylon 10I, nylon 10I/10T and nylon 6–3-T. The TGA and DTG curves of nylon 10I, nylon 10I/10T and nylon 6–3-T are presented in Figures 6(a,b,c) and 7(a,b,c) respectively. The relevant parameters are summarized in Table 5. Among them, *T_1_* stands for the extrapolated onset temperature, which refers to the start of the reaction, *T_m_* represents the temperature where the highest degradation rate occurred and *T_2_* refers to the extrapolated end temperature. It can be observed that the *T_1_* of nylon 10I and nylon 10I/10T are both higher than nylon 6–3-T (Table 5). These results indicate that both nylon 10I and nylon 10I/10T have better thermal stability than nylon 6–3-T. In the DTG curves, the minimum values of d*α*/d*t* corresponding to the temperatures of the samples’ maximum degradation rate. With increasing heating rate, the *T_1_, T_m_* and *T_2_* have shifted towards a higher temperature, but the maximum rate of degradation decreased. This temperature-induced change may be contributed to the heat transfer. At lower heating rates, the heat transfer was more effective than it was at higher heating rates. The DTG profiles for the samples of nylon 10I and nylon10I/10T, tested in N_2_, reveal a single peak within the temperature range of 400–500 °C, which indicates that the major mass loss took place in one-step. Furthermore, the addition of PTA did not influence the thermal decomposition pattern of nylon 10I. In addition, Table 5 presents that the *T_1_, T_m_* and *T_2_* values of nylon 10I/10T are higher than nylon 10I due to enhanced intermolecular force.

**Table 5.** The TGA and DTG data of nylon 10I, nylon 10I/10T and nylon 6–3-T.

Samples	Heating rate(°C/min)	T_1_	T_m_	T_2_
nylon 10I	5	429.78	465.26	473.67
	10	445.67	478.41	491.64
	20	461.56	488.42	509.40
	30	469.70	492.61	517.55
nylon 10I/10T	5	438.00	474.29	483.47
	10	457.06	484.46	498.77
	20	468.38	495.10	513.85
	30	475.92	509.88	528.90
nylon 6–3-T	5	405.02	425.50	433.86
	10	412.19	434.43	446.14
	20	426.89	447.39	462.42
	30	435.82	454.20	471.40

### Kinetics analysis of the decomposition process

#### Theoretical background

The general expression for the kinetic description of the degradation of solid material is given below:

(1) $$B_{solid}\;=\;C_{solid}+D_{gas}$$

Where *B_solid_* represents the starting material, *C_solid_* refers to the solid product and *D_gas_* corresponds to the gaseous product during the disappearance of *B_solid_* [27]. The TGA experiment results can be expressed as a function of the extent of reaction *α*, which corresponds to the degree of decomposition.

(2) $$\alpha \;=\;\frac{W_{0}\;-\;W_{t}}{\;W_{0}\;-\;W_{f}}$$

where *W_t_, W_0_* and *W_f_* correspond to the actual, initial and final mass of the sample, respectively.

The disappearance rate, d*α*/d*t*, can be expressed as:

(3) $$\frac{d_{\alpha }}{d_{t}}=k\left(T\right)f\left(\alpha \right)$$

where *k*(*T*) corresponds to the reaction rate constant and *f(α)* refers to the differential conversion function.

After substitution of the Arrhenius equation, the conversion degree can be analyzed as a function of temperature as below:

(4) $$k\left(T\right)=Aexp\left(\frac{E}{RT}\right)$$

where *A* represents the pre-exponential factor, *E* refers to the apparent activation energy, *T* corresponds to the temperature and *R* represents the gas constant.

By combining Eq. (3) and (4), we can obtain

(5) $$\frac{\;d_{\alpha }}{d_{t}}=Aexp\left(-\frac{E}{RT}\right)f\left(\alpha \right)$$

Under non-isothermal conditions, the kinetics analysis is performed as [28]:

(6) $$\frac{d_{\alpha }}{d_{T}}=\frac{A}{\beta }exp\left(-\frac{E}{RT}\right)f\left(\alpha \right)$$

where (*β = *d*T/*d*t*) represents the constant heating rate.

After re-arranging the variables, we obtain:

(7) $$g\left(\alpha \right)=\int_{0}^{\alpha }\;\frac{d_{\alpha }}{\int \left(\alpha \right)}=\int_{T_{0}}^{T_{P}}\frac{A}{\beta }\;exp\left(-\frac{E}{RT}\right)dT=\frac{AE}{\beta T}P\left(\chi \right)$$

where *χ* is equal to *E/RT*.

In this paper, different mathematical methods were used to explain the previous equation.

#### KAS method

Usually, iso-conversional integral methods are used to provide an apprehensible activation energy values [29]. The KAS method is an integral method, suggested by Coats and Redfern [30], based on the approximation of $p\;\left(\chi \right)≅\chi^{-2}{e^{-}}^{\chi }$ and the obtained equation can be given as follow:

(8) $$ln\frac{\beta }{T^{2}}=ln\frac{AR}{Eg\left(\alpha \right)}-\frac{E}{RT}$$

The activation energy can be determined from the slope of ln(*β/T^2^*) versus 1/*T_p_* plot. The correct kinetic model was found by the value of the activation energy using Coats–Redfern method.

**Figure 8.**
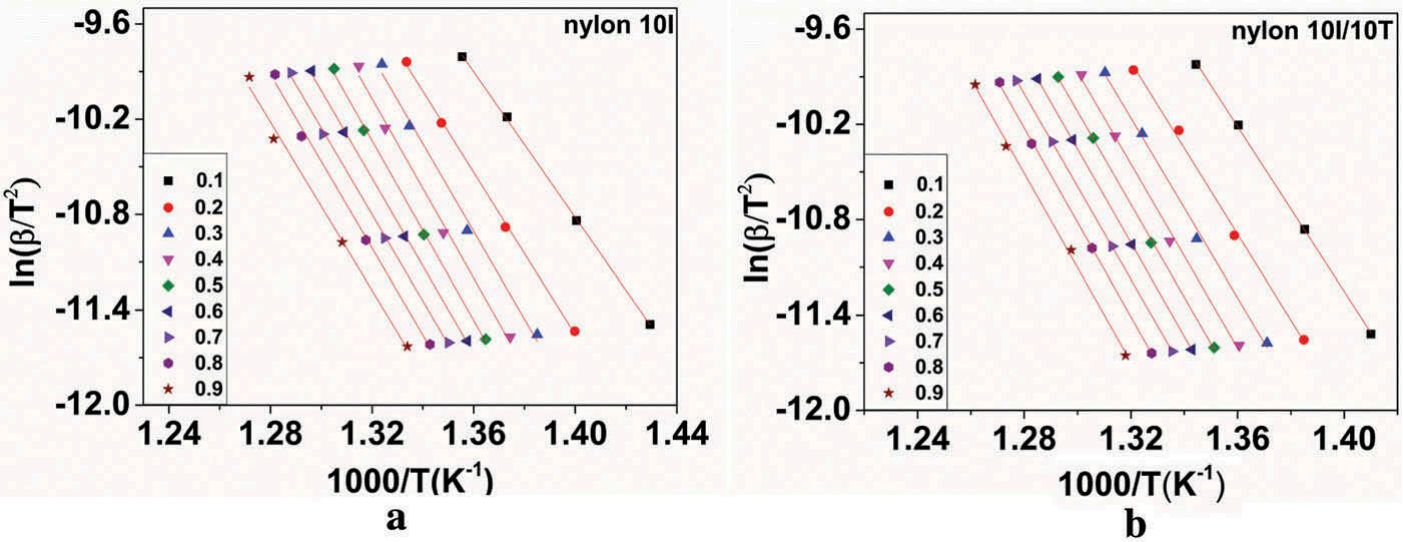
Isoconversional curves of the KAS method: (a) nylon 10I and (b) nylon 10I/10T.

According to Eq. 8, the KAS method was used to calculate the activation energy of nylon 10I and nylon 10I/10T at different conversion rates under N_2_ atmosphere. The above results are described in Figure 8(a,b), respectively. Both figures have shown a series of parallel lines, which indicates that the reaction mechanism did not change during the decomposition process. From the slopes of the plots in Figure 8(,b), the activation energy of nylon 10I and nylon 10I/10T were obtained as *E* (nylon 10I) = -average slope*R = 222.86 kJ/mol and *E* (nylon 10I/10T) = average slope*R = 238.00 kJ/mol, respectively.

#### FWO method

The FWO method is also an integral method and can be used to estimate the apparent activation energy without precise reaction mechanism. This method is based on Doyle’s approximation [31].

By replacing Doyle’s approximation into Eq. (7):

(9) $$log\beta =log\frac{AE}{g\left(\alpha \right)R}-2.315-\frac{0.4567E}{RT}$$

**Figure 9.**
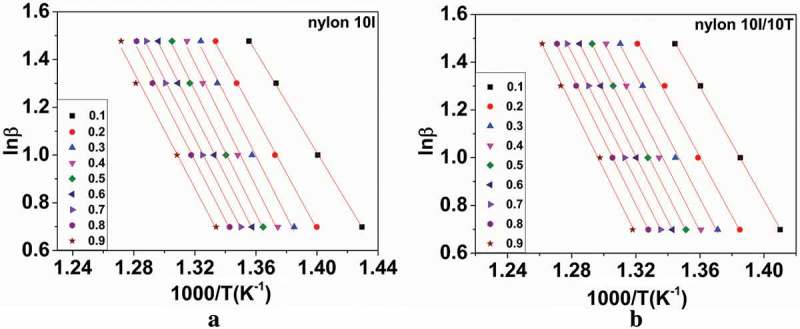
Isoconversional curves of the FWO method: (a) nylon 10I and (b) nylon 10I/10T.

Figure 9(a,b) show the FWO analysis of nylon 10I and nylon 10I/10T, under the N_2_ atmosphere, with conversion range of 0.1–0.9, respectively. It can be seen from Figure 9(a,b) that the slopes of lines are almost constant in the conversion range of 0.1–0.9, which indicate that the reaction mechanisms are unchanged. The average activation energies of nylon 10I and nylon10I/10T calculated by FWO method are 223.74 and 238.25 kJ/mol, respectively. It can be observed that the apparent *E* values calculated by the FWO method were in perfect accordance with the KAS method.

#### Tang method

According to the Eq. (7), taking the logarithms of both sides, we can obtain:

(10) $$\begin{array}{rl} & ln\left(\frac{\beta }{T^{1.894661}}\right)=ln\left(\frac{AE}{Rg\left(\alpha \right)}\right)+3.635041-1.894661\;lnE\\ & \;\;\;\;\;\;\;\;\;\;\;\;\;\;\;\;\;\;\;\;\;\;\;\;\;\;\;\;\;\;\;\;\;-\frac{1.001450E}{RT} \end{array}$$

**Figure 10.**
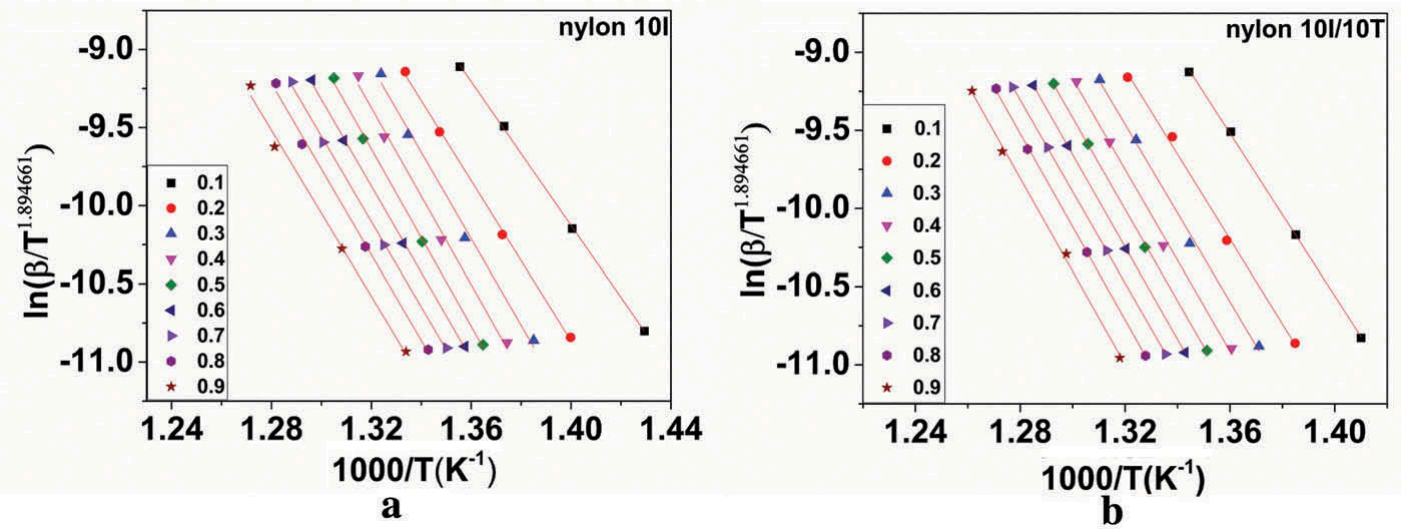
Isoconversional curves of the Tang method: (a) nylon 10I and (b) nylon 10I/10T.

The activation energy can be calculated from the slope of ln(*β*/*T*^1.894661^) versus 1/*T* plots [32]. The plots between the activation energy and reaction extent of nylon 10I and nylon 10I/10T, under the N_2_ atmosphere, are presented in Figure 10(a,b), respectively. Next, we calculated the average slope and activation energy for nylon 10I and nylon 10I/10T as below: Average slope = −1.001450*E*/*R, E* (nylon 10I) = 223.52 KJ/mol and *E* (nylon 10I/10T) = 238.65 KJ/mol.

Similar values of activation energy were obtained by the KAS, FWO and Tang methods, which indicates that these three methods are suitable for the calculation of activation energy of nylon10I and nylon 10I/10T. The values of activation energy of nylon10I and nylon 10I/10T calculated by KAS, FWO and Tang methods are listed in Tables 6 and 7, respectively. Furthermore, one should note that the activation energy values of nylon 10I/10T are higher than the nylon 10I, which corresponds to the enhanced heat resistance of nylon 10I/10T.

**Table 6.** Activation energies of nylon 10I calculated by KAS, FWO and Tang methods.

Conversion	KAS method		FWO method		Tang method	
	E(KJ mol^−1^)	R^2^	E(KJ mol^−1^)	R^2^	E(KJ mol^−1^)	R^2^
0.1	190.71	0.9995	192.72	0.9996	191.34	0.9995
0.2	212.34	0.9985	213.49	0.9986	212.98	0.9985
0.3	229.31	0.9906	229.73	0.9914	229.96	0.9906
0.4	234.78	0.9918	235.04	0.9926	235.43	0.9919
0.5	235.07	0.9981	235.40	0.9983	235.73	0.9981
0.6	229.61	0.9991	230.25	0.9992	230.27	0.9991
0.7	226.52	0.9987	227.40	0.9988	227.18	0.9987
0.8	227.19	0.9947	228.08	0.9952	227.86	0.9947
0.9	220.21	0.9914	221.54	0.9923	220.88	0.9914
mean	222.86	-	223.74	-	223.52	-

**Table 7.** Activation energies of nylon 10I/10T calculated by KAS, FWO and Tang methods.

Conversion	KAS method		FWO method		Tang method	
	E(KJ mol^−1^)	R^2^	E(KJ mol^−1^)	R^2^	E(KJ mol^−1^)	R^2^
0.1	215.77	0.9995	216.67	0.9996	216.41	0.9995
0.2	225.15	0.9949	225.79	0.9954	225.79	0.9949
0.3	235.29	0.9952	235.54	0.9957	235.94	0.9953
0.4	240.73	0.9945	240.79	0.9950	241.39	0.9945
0.5	242.96	0.9994	243.00	0.9994	243.63	0.9994
0.6	245.04	1.0000	245.06	1.0000	245.71	1.0000
0.7	242.97	1.0000	243.16	1.0000	243.64	1.0000
0.8	247.06	0.9996	247.11	0.9996	247.74	0.9996
0.9	246.99	0.9974	247.14	0.9976	247.67	0.9974
mean	238.00	-	238.25	-	238.65	-

Unlike other methods, the KAS, FWO and Tang methods can be used to obtain the activation energies without the prior knowledge of the reaction mechanism [33]. To the best of our knowledge, different mathematical methods have been reported in the literature to validate the thermal degradation mechanism models by comparing the activation energies obtained from the ‘model-free’ methods [34].

#### Coats–Redfern method

The Coats-Redfern, the most common ‘model-free’ method, uses an asymptotic approximation and rearranges Eq. (7) at different conversion values [35]. By substituting the ln(1-2*RT/E*)→0, the following equation can be obtained [36]:

(11) $$ln\frac{g\left(\alpha \right)}{T^{2}}=ln\frac{AR}{\beta E}-\frac{E}{RT}$$

According to the different degradation processes, with the algebraic expression of *g*(*α*) for different kinetic mechanisms (Table 8), we can obtain the activation energy and the correlation coefficient from the ln[*g*(*α*)/*T^2^*] versus 1/*T* plot, as well as the valid reaction mechanism [37].

**Table 8.** Algebraic expressions for g(α) for the most frequently used mechanisms of solid-state processes.

Symbol	g(α)	Solid-state processes
Sigmoidal Curves		
A_2_	[-ln(1-α)]^1/2^	Nucleation and growth [Avrami–Erofeev, eq. (1)]
A_3_	[-ln(1-α)]^1/3^	Nucleation and growth [Avrami–Erofeev, eq. (2)]
A_4_	[-ln(1-α)]^1/4^	Nucleation and growth [Avrami–Erofeev, eq. (3)]
Decleration Curves		
R_1_	α	Phase-boundary-controlled reaction (one-dimensional movement
R_2_	1−(1−α)^1/2^	Phase-boundary-controlled reaction (contracting area)
D_1_	α^2^	One-dimensional diffusion
D_2_	（1-α)ln(1-α)+ α	Two-dimensional diffusion (Valensi equation)
D_3_	[1-(1-α)^1/3^]^2^	Three-dimensional diffusion (Jander equation)
D_4_	(1-2α/3)-(1-α)^2/3^	Three-dimensional diffusion (Ginstling–Brounshtein equation)
Acceleration curves		
P2	α^1/2^	Random nucleation with one nucleus on the individual particle
P3	α^1/3^	Random nucleation with two nuclei on the individual particle
P4	α^1/4^	Random nucleation with two nuclei on the individual particle

Compared to the results calculated by the KAS, FWO, and Tang methods, the smaller deviation represents better consistency with the linear relationship equation. It is usually expressed by the correlation coefficient *R^2^*, which indicates that the function *g*(*α*) reflects the real situation.

The thermal decomposition activation energy values and correlation coefficients of nylon 10I and nylon 10I/10T from the Coats–Redfern plots are listed in Tables 9 and 10, respectively. The values of kinetic parameters vary with respect to the change in the reaction mechanism. In addition, the heating rate exerts a significant effect on the values of the kinetic parameters, which is consistent with the previously published literature [20]. Moreover, the *E* values, calculated by KAS, FWO and Tang methods, of nylon 10I and nylon 10I/10T correspond to the R2 mechanism at the heating rate of 5 and 20 °C/min, respectively. Taken together, the kinetics analysis implies that the thermal degradation of nylon 10I and nylon 10I/10T follow the phase boundary-controlled reaction type, which is referred as an R2 mechanism.

**Table 9.** Activation energies of nylon 10I calculated by Coats–Redfern method at the heating rate of 5, 10, 20 and 30 °C/min.

Mechanism	5°C/min		10°C/min		20°C/min		30°C/min	
	E(KJ mol^−1^)	R^2^	E(KJ mol^−1^)	R^2^	E(KJ mol^−1^)	R^2^	E(KJ mol^−1^)	R^2^
A2	123.12	0.9895	129.13	0.9916	129.98	0.9980	139.87	0.9972
A3	78.07	0.9885	81.99	0.9908	82.47	0.9978	89.02	0.9970
A4	55.54	0.9874	58.42	0.9899	58.72	0.9976	63.60	0.9966
R1	185.60	0.9933	194.00	0.9890	193.68	0.9746	207.27	0.9652
R2	218.49	0.9984	228.60	0.9971	229.24	0.9929	245.68	0.9880
D1	383.24	0.9937	400.28	0.9897	399.91	0.9763	427.21	0.9673
D2	423.28	0.9979	442.37	0.9955	443.07	0.9878	473.84	0.9816
D3	473.96	0.9973	495.76	0.9971	498.07	0.9964	533.26	0.9932
D4	439.94	0.9985	459.92	0.9969	461.15	0.9915	493.37	0.9864
P2	86.77	0.9922	90.85	0.9874	90.57	0.9709	97.30	0.9605
P3	53.83	0.9909	56.47	0.9854	56.20	0.9662	60.64	0.9547
P4	37.36	0.9893	39.28	0.9829	39.02	0.9605	42.31	0.9477

**Table 10.** Activation energies of nylon 10I/10T calculated by Coats–Redfern method at the heating rate of 5, 10, 20 and 30 °C/min.

Mechanism	5°C/min		10°C/min		20°C/min		30°C/min	
	E(KJ mol^−1^)	R^2^	E(KJ mol^−1^)	R^2^	E(KJ mol^−1^)	R^2^	E(KJ mol^−1^)	R^2^
A2	127.53	0.9981	135.23	0.9917	134.82	0.9994	143.24	0.9980
A3	80.95	0.9979	86.02	0.9910	85.66	0.9994	91.24	0.9978
A4	57.66	0.9977	61.41	0.9902	61.09	0.9993	65.23	0.9976
R1	190.72	0.9839	203.07	0.9908	201.02	0.9795	213.22	0.9775
R2	225.30	0.9977	239.16	0.9981	237.70	0.9961	252.07	0.9945
D1	393.64	0.9849	418.56	0.9914	414.67	0.9808	439.22	0.9789
D2	435.68	0.9941	462.46	0.9968	459.24	0.9915	486.41	0.9898
D3	489.07	0.9995	518.10	0.9978	515.93	0.9988	546.44	0.9974
D4	453.23	0.9968	480.75	0.9979	477.88	0.9948	506.14	0.9932
P2	89.26	0.9814	95.33	0.9894	94.19	0.9765	100.22	0.9745
P3	55.44	0.9785	59.42	0.9878	58.58	0.9728	62.56	0.9707
P4	38.53	0.9747	41.46	0.9858	40.78	0.9683	43.73	0.9662

## Conclusions

Herein, we have synthesized a novel bio-based transparent nylon 10I/10T by copolymerization modification. The FT-IR and ^1^H NMR confirmed the chemical structure of nylon 10I/10T, which possess high intrinsic viscosity, excellent mechanical properties and solvent resistance. Furthermore, we have observed a glass transition temperature of 118.9°C for nylon 10I/10T. Interestingly, as-prepared nylon 10I/10T has shown much lower water absorption of 0.23% than nylon 6–3-T (7.5%).

The TGA results show that thermal decomposition onset temperature of nylon 10I/10T (438 °C) is higher than the thermal decomposition onset temperature of (nylon 6-3-T (405.02 °C), which confirms the excellent thermal stability of nylon 10I/10T. The KAS, FWO, Tang and Coats–Redfern methods were used to determine the activation energy and reaction mechanism of nylon 10I/10T which shows that the thermal degradation of nylon 10I/10T follows the R2 type reaction mechanism. These results present the promising applicability of bio-based transparent nylon 10I/10T in the development of the candidate plastics for the industrial production.

## Supplementary material

Supplementary data cane be accessed here.
