# Supplementary material for: Synthesis, characterization and thermal decomposition kinetics of a bio-based transparent nylon 10I/10T
Source: Des Monomers Polym. 2018 Nov 14;21(1):182–92. doi: 10.1080/15685551.2018.1543633 (PMC6237164; doi:10.1080/15685551.2018.1543633)
Supplement: Supplemental Material [file TDMP_A_1543633_SM5078.docx]

***Supporting Information***

**Synthesis, characterization and thermal decomposition kinetics of bio-based transparent nylon 10I/10T**

Bingxiao Liu; Guosheng Hu; Jingting Zhang; Chunhui Fang

**Figure of contents**

1. The reaction scheme of nylon10I/10T ·····················································S1

1. The reaction scheme of nylon10I/10T





Figure. S1 The raction scheme of nylon 10I/10T
